# Primary osteogenic sarcoma of the sternum with intravascular invasion and rapid progression: a rare case report

**DOI:** 10.1097/MS9.0000000000005065

**Published:** 2026-04-30

**Authors:** Aleeza Abid, Mubashira Noor, Abdullah Saad, Solay Farhat, Nazish Sikander, Tanveer Ahmad

**Affiliations:** a Department of Thoracic Surgery, Sindh Medical College, Jinnah Sindh Medical University, Karachi, Pakistan; b Faculty of Medical Sciences, Lebanese University, Beirut, Lebanon; c Department of Thoracic Surgery, Jinnah Postgraduate Medical Centre, Karachi, Pakistan

**Keywords:** case report, intravascular invasion, primary sternal osteosarcoma

## Abstract

**Introduction and importance:**

Primary osteosarcoma of the sternum is a rare malignant bone tumor. Only a few cases have been documented, and most have been managed with chemotherapy and surgical intervention. Our case is distinctive due to the vascular invasion of the left brachiocephalic vein and lack of response to chemotherapy. Reporting this case contributes to the limited literature and highlights the diagnostic and therapeutic challenges associated with the management of primary sternal osteosarcoma.

**Case presentation:**

We describe a 37-year-old woman with a painful, progressively enlarging manubrial swelling. Imaging revealed a destructive sternal mass with mediastinal extension, pulmonary and nodal metastases, and a thrombus extending from the left brachiocephalic vein to the superior vena cava. Magnetic resonance imaging confirmed soft tissue infiltration, cardiac compression, and worsening pericardial effusion. Biopsy revealed malignant osteoid-forming cells with SATB2 positivity, confirming the diagnosis of primary sternal osteosarcoma. She received three cycles of doxorubicin and cisplatin, but her condition rapidly progressed. Regorafenib was initiated as salvage therapy; however, her condition worsened, and she died within 6 months of diagnosis.

**Clinical discussion:**

Primary osteosarcoma of the sternum is a rare and aggressive malignancy in adults. Imaging is crucial for staging and planning surgery. Standard treatment includes neoadjuvant chemotherapy with wide excision and reconstruction, whereas targeted therapies such as regorafenib may be considered for metastatic diseases.

**Conclusion:**

This case illustrates an exceptionally aggressive tumor complicated by vascular invasion and mediastinal involvement. This underscores the importance of early recognition to avoid diagnostic delays and demonstrates the limited efficacy of conventional chemotherapy in advanced disease. The use of regorafenib highlights the emerging systemic options that may be explored in refractory osteosarcoma. Our report emphasizes the urgent need for early diagnostic practices and a multidisciplinary approach to treatment.

## Introduction

Primary osteogenic sarcoma (osteosarcoma) of the sternum is an extremely rare and aggressive malignant bone tumor with a poor prognosis[1]. Osteosarcoma arises from primitive mesenchymal cells capable of producing osteoid (hallmark malignant bone matrix). It typically affects metaphyseal regions of long bones – most commonly the distal femur, proximal tibia, and proximal humerus – and less frequently the pelvis. Although it is the most common primary malignant bone tumor in children and adolescents, its occurrence in flat bones such as the sternum is extremely uncommon and usually presents in adults. Histologically, osteosarcoma can be classified into osteoblastic, chondroblastic, and fibroblastic subtypes[2].

HIGHLIGHTS

- Primary sternal osteosarcoma is extremely rare with few reported cases.
- This case shows intravascular invasion into the left brachiocephalic vein.
- The tumor progressed rapidly and was resistant to first-line chemotherapy.
- Regorafenib was used as salvage therapy for refractory osteosarcoma.
- Highlights diagnostic challenges and need for multidisciplinary management.

Our case is distinct for several reasons. First, the tumor showed direct intravascular extension into the left brachiocephalic vein – an occurrence rarely reported in the existing literature. Second, it produced a substantial mediastinal mass effect, resulting in progressive pericardial effusion, a consequence rarely seen in association with osteosarcoma of the sternum. Finally, the tumor demonstrated no response to chemotherapy and continued to increase in size despite treatment. Reporting this case contributes to the limited literature and highlights the diagnostic and therapeutic challenges associated with managing primary sternal osteosarcoma. The ethical disclosure and transparency regarding the use of artificial intelligence in manuscript preparation were ensured in accordance with the TITAN Guideline Checklist 2025 for AI Reporting in Research[3]. Adetailed chronological overview of the patient’s clinical course issummarized in Table 1.

**Table 1:** Timeline of case report.

Time	Clinical course
3 months prior	Progressive painful swelling over the sternum
At presentation	Hard presternal mass; anemia and elevated alkaline phosphatase
Initial imaging	CT/MRI showed large sternal mass with mediastinal extension, intravascular invasion, pulmonary metastases, and pericardial effusion
Diagnosis	Core biopsy confirmed primary osteoblastic osteosarcoma
Treatment	Three cycles of neoadjuvant chemotherapy (doxorubicin and cisplatin)
Reassessment	Imaging showed rapid disease progression
Further management	Regorafenib initiated
Outcome	Death due to progressive disease within 6 months

## Timeline

## Case presentation

A 37-year-old woman presented to the department of thoracic surgery with a 3-month history of painful, progressively enlarging swelling over the manubrium of the sternum. Patient had no prior history of trauma, exposure to radiation, or family history of malignancy. Initially, it was misdiagnosed to be breast carcinoma.

On physical examination, the mass appeared hard and tender without any overlying skin changes. Laboratory investigations revealed mild anemia (hemoglobin 10.2 g/dL) and increased levels of gamma-glutamyl transferase and alkaline phosphatase, with all other parameters being in the normal range.

**Figure 1.**
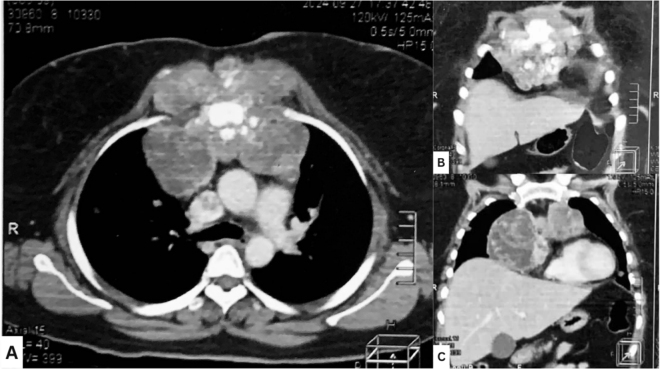
CT scan pre-chemotherapy. (A) Transverse cut. (B, C) Coronal cut demonstrating the extension of the mass, involving the presternal, parasternal, and retrosternal regions of the chest wall.

A chest computerized tomography scan (CT scan) revealed a large solid-cystic mass in the sternum, measuring 11.6 × 12.8 × 10.2 cm, extending into presternal, parasternal, and retrosternal regions. The mass displaced the heart toward the left side, leading to mild pericardial effusion and extensive erosion of the sternum and adjacent chondrosternal joints. Additional findings included right hilar lymphadenopathy measuring 3.3 × 3.2 cm, pulmonary metastases, and a hypodense intravascular thrombus within the left brachiocephalic vein extending into the superior vena cava. Magnetic resonance imaging (MRI) confirmed an infiltrative neoplasm with exophytic soft tissue components extending into the subcutaneous fat (Fig. 1, pre-chemotherapy).

Histopathological analysis of the obtained core biopsy sample revealed multiple linear cores with a cellular neoplastic lesion. The cells showed moderate cytoplasm, round to irregular hyperchromatic nuclei, marked pleomorphism, and frequent mitotic activity. A prominent lacy deposition of malignant osteoid was also seen. Immunohistochemical examination displayed positivity for SATB2, supporting the diagnosis of primary osteoblastic osteogenic sarcoma of the sternum.

**Figure 2.**
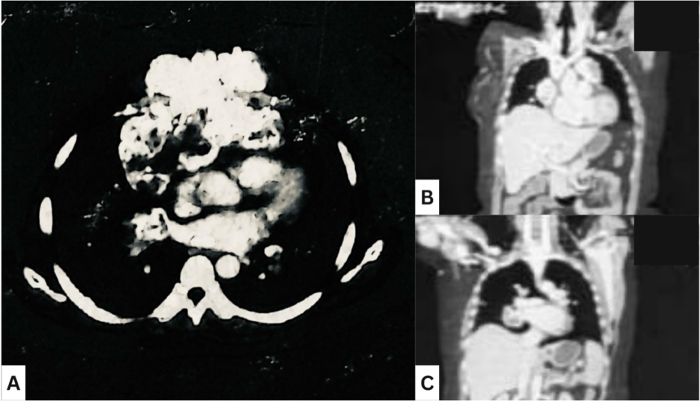
CT scan post-chemotherapy. (A) Transverse cut. (B, C) Coronal cut post-chemotherapy.

The patient received three cycles of neoadjuvant chemotherapy, which comprised doxorubicin (30 mg) and cisplatin (30 mg) administered intravenously every 3 weeks, along with ondansetron (8 mg) for antiemetic prophylaxis. A follow-up CT scan showed rapid disease progression, with the mass increasing in size to 10.0 × 15.8 × 15.3 cm, with increased retrosternal and intravascular extension into the left brachiocephalic vein, enlargement of the right hilar lymph node to 5.7 × 4.3 cm, and the number and size of pulmonary metastases also increased significantly, with the largest pleural-based nodule measuring 2.4 × 1.6 cm. The pericardial effusion increased slightly, and a small cystic lesion was also observed in the right lobe of the liver in the hepatic segment VIII (5 × 3 mm) (Fig. 2, post-chemotherapy).

Due to the aggressive progression of the disease, it was evaluated in a multidisciplinary setting that included thoracic and plastic surgeons. Surgical resection was considered unfeasible because of the patient’s deteriorating condition and the high risk of intraoperative hemorrhage. Consequently, the patient was started on oral regorafenib (150 mg daily for 21 days followed by a 1-week gap). Despite this targeted therapy, her clinical condition continued to worsen, and she succumbed to the disease within 6 months of her diagnosis.

## Discussion

Osteosarcoma is a highly aggressive malignant bone tumor arising from mesenchymal cells, capable of producing osteoid, and is commonly recognized as associated with particular genetic predispositions. Germline mutations in RB1, TP53 (Li-Fraumeni syndrome), RECQL4, BLM, and WRN increase the likelihood of osteosarcoma[4]. No predisposing genetic syndrome or risk factor was identified in our patient, showing the unpredictable nature of this rare entity. Osteosarcoma exhibits two distinct peaks in accordance with age, with a primary peak in adolescence, predominantly affecting long bones, and a secondary peak in adults over 50 years of age, often involved with extra skeletal or atypical presentations^[^5,6^]^. Primary osteosarcoma of the sternum, as in this case, represents a distinct clinical entity due to its rarity and adult onset.

Imaging is essential in diagnosing, staging, and treatment planning of this disease. CT scans are useful in demonstrating the effect of osteosarcoma on bone – whether destructive, osteogenic, or mixed. They clearly show bone destruction and calcification[5]. However, MRI provides superior detail when assessing the extent of soft tissue and bone marrow involvement, which is particularly important for tumors extending to mediastinal structures or vascular channels. Although radiological-guided True-cut needle biopsy may be used for diagnosis, an open biopsy under anesthesia is recommended to obtain sufficient tissue for comprehensive histopathological and immunohistochemical analyses[7].

Treatment for primary osteosarcoma of the sternum typically involves neoadjuvant chemotherapy along with surgical resection and reconstruction. In developed countries, methotrexate, cisplatin, and doxorubicin, known as MAP chemotherapy, are frequently used as neoadjuvant chemotherapy. While non-methotrexate-based regimens, frequently used in Asian populations, have also demonstrated a favorable response rate of 58% in treatment of osteosarcoma. Studies have found that fibroblastic and osteoblastic subtypes exhibit a more favorable response to chemotherapy than chondroblastic subtypes[8].

Various reconstructive methods have been described, including the use of synthetic materials like Marlex mesh, Gore-Tex, and myocutaneous flaps^[^2,9^]^. While surgical excision combined with neoadjuvant and adjuvant chemotherapy is the primary treatment for sternal malignancies[10], surgical excision may not be feasible in patients with metastasis or cardiopulmonary impairment, as they are at high risk of developing adverse postoperative complications; however, chemotherapy can still be administered in such cases[2]. Regorafenib, a multikinase inhibitor, is used as a second line option in patients with relapsed, refractory and metastatic osteosarcoma. In a randomized study, 65% of patients with progressive, metastatic osteosarcoma, unresponsive to chemotherapy, treated with regorafenib did not show disease progression at 8 weeks, compared to none in the placebo group[11]. Despite receiving two cycles of this targeted therapy, our patient failed to exhibit a favorable response.

What sets our case apart from other reported sternal osteosarcomas are several uncommon clinical and radiological features. First, the tumor displayed direct intravascular extension into the left brachiocephalic vein – a phenomenon rarely reported in existing literature. Intravascular invasion is extremely uncommon and carries a high risk of tumor thrombus, pulmonary embolism, and hemodynamic compromise. Additionally, the mass exerted significant mediastinal compression, causing progressive pericardial effusion. Although mediastinal extension has been occasionally reported in previous case reports of sternal osteosarcomas, associated pericardial effusion remains scarcely described in the existing literature. Finally, the tumor exhibited no response to chemotherapy and targeted therapy (Regorafenib), with continued progression despite standard neoadjuvant therapy, underscoring its highly aggressive biological behavior. This lack of response must be interpreted with caution, as the patient exhibited rapidly progressive disease, extensive vascular invasion, and declining clinical status, all of which may have limited the potential therapeutic benefit of targeted therapy. Consequently, conclusions regarding the efficacy of chemotherapy and regorafenib in sternal osteosarcoma cannot be drawn from this single case.

A comparison with published cases further highlights the unique nature of this presentation. Among reported primary chest wall osteosarcomas, patients without metastatic disease who underwent wide surgical excision with chemotherapy were generally associated with favorable outcomes. For example, a 60-year-old man with fibroblastic osteosarcoma limited to the chest wall showed good survival after undergoing two-staged resections for recurrent disease[2], while a 57-year-old man with mediastinal extension remained recurrence-free after chemotherapy and surgical excision[5]. Conversely, patients presenting with metastatic disease treated only with chemotherapy revealed rapid progression and poor survival, as observed in a 55-year-old man with osteoblastic osteosarcoma and pulmonary metastases who died within 6 months of diagnosis[12]. In a younger patient, a 24-year-old male with chondroblastic osteosarcoma, end results were favorable when treatment included preoperative chemotherapy followed by surgery[10]. Other cases highlight different end results depending on tumor extension, cardiopulmonary involvement, and the feasibility of surgical excision^[^1,13,14^]^. Table 2 summarizes the literature on primary osteosarcoma of the sternum.

**Table 2:** Summary of existing case reports on primary osteosarcoma of sternum.

Age	Gender	Type/pathology	Tumor extension	Treatment	Survival	Reference
60 years	Male	Fibroblastic osteosarcoma	No cardiopulmonary extension	Surgical resection twice, with a gap of 8 months as the neoplasm relapsed	The patient survived well	[2]
57 years	Male	Unspecified osteosarcoma	Extending anteriorly and posteriorly into the mediastinum	Surgical resection along with pre- and postoperative chemotherapy and radiotherapy	No recurrence was observed	[5]
55 years	Male	Osteoblastic osteosarcoma	Pulmonary metastasis along with extension into retrosternal anterior mediastinum	Chemotherapy	The patient died within 6 months of diagnosis	[12]
24 years	Male	Chondroblastic osteosarcoma	Involving seventh and eighth right rib cartilage	Preoperative chemotherapy along with surgical resection	No recurrence in 19 months and resulted in good health	[10]
60 years	Female	Fibroblastic + osteoblastic osteosarcoma	Extension into upper mediastinum, displacing innominate vein without infiltrating it. Also involving pectoral muscles and mild renal and cardiac insufficiency was also observed	Surgical excision with postoperative chemotherapy	No recurrence after 16 months follow-up	[10]
36 years	Male	Chondroblastic osteosarcoma	Extension into anterior mediastinum, involving pectoral muscles. Pulmonary, pleural, and pericardial effusions partially calcified aorticopulmonary, right hilar and subcardinal lymphadenopathy was also observed	Chemotherapy	Not mentioned	[1]
74 years	Male	Unspecified osteosarcoma	No cardiopulmonary extension	Surgery and adjuvant radiotherapy	No recurrence within 2 years	[13]
71 years	Male	Unspecified osteosarcoma	Extension into anterior mediastinum and also involving the pectoralis muscle	Surgery	Not mentioned	[14]

To our knowledge, this combination of aggressive behavior, vascular invasion, and no response of the tumor to conventional chemotherapy and targeted therapy has not been described in previous sternal osteosarcoma case reports, making this case uniquely informative for both clinicians and researchers.

## Conclusion

Herein, we report a case of primary osteosarcoma of the sternum. Although osteosarcoma is the most common primary malignant bone tumor, accounting for approximately 20% of all cases, its occurrence in the sternum is extremely rare. The 5-year survival rate is very poor, approximately 15%, which decreases further in cases of metastatic disease[9]. Given the poor prognosis and poor survival rate of this rare disease despite surgery and chemotherapy, there is limited literature available on it. Our patient’s diagnosis was delayed, and, unfortunately, the tumor continued to progress, which led to a much deteriorated condition, created significant challenges for further treatment, and resulted in a very short survival.

## Methods/search strategy

Study design: This was a case report (retrospective, single-center clinical case report). A narrative literature search was performed using PubMed/Medline and Google Scholar for published case reports and series of primary osteosarcoma of the sternum up to October 2025. Search terms included “sternum,” “osteosarcoma,” “primary bone cancers,” and “case report.” Articles in English were reviewed, and references within identified papers were screened for additional cases to compare clinical presentation, management, and outcomes. This case report is prepared in accordance with CARE guideline.

## Ethical approval

The case was explained to the patient’s guardian both verbally and in writing. Written informed consent for publication was obtained from the patient’s guardian.

## Data Availability

The data supporting the findings of this study are available from the corresponding author upon reasonable request.
